# A Simultaneous Localization and Mapping System Using the Iterative Error State Kalman Filter Judgment Algorithm for Global Navigation Satellite System

**DOI:** 10.3390/s23136000

**Published:** 2023-06-28

**Authors:** Bo You, Guangjin Zhong, Chen Chen, Jiayu Li, Ersi Ma

**Affiliations:** 1 Heilongjiang Provincial Key Laboratory of Complex Intelligent System and Integration, Harbin University of Science and Technology, Harbin 150080, China; youbo@hrbust.edu.cn (B.Y.); 2120510126@stu.hrbust.edu.cn (G.Z.); lijiayu@hrbust.edu.cn (J.L.); maersi97@163.com (E.M.); 2 Key Laboratory of Intelligent Technology for Cutting and Manufacturing Ministry of Education, Harbin University of Science and Technology, Harbin 150080, China

**Keywords:** autonomous mobile robots, Global Positioning System (GPS), LiDAR, inertial measurement unit (IMU), Iterative Error State Kalman Filter (IESKF), Simultaneous Localization and Mapping (SLAM)

## Abstract

Outdoor autonomous mobile robots heavily rely on GPS data for localization. However, GPS data can be erroneous and signals can be interrupted in highly urbanized areas or areas with incomplete satellite coverage, leading to localization deviations. In this paper, we propose a SLAM (Simultaneous Localization and Mapping) system that combines the IESKF (Iterated Extended Kalman Filter) and a factor graph to address these issues. We perform IESKF filtering on LiDAR and inertial measurement unit (IMU) data at the front-end to achieve a more accurate estimation of local pose and incorporate the resulting laser inertial odometry into the back-end factor graph. Furthermore, we introduce a GPS signal filtering method based on GPS state and confidence to ensure that abnormal GPS data is not used in the back-end processing. In the back-end factor graph, we incorporate loop closure factors, IMU preintegration factors, and processed GPS factors. We conducted comparative experiments using the publicly available KITTI dataset and our own experimental platform to compare the proposed SLAM system with two commonly used SLAM systems: the filter-based SLAM system (FAST-LIO) and the graph optimization-based SLAM system (LIO-SAM). The experimental results demonstrate that the proposed SLAM system outperforms the other systems in terms of localization accuracy, especially in cases of GPS signal interruption.

## 1. Introduction

With the continuous development of outdoor mobile robots, research focuses mainly on two directions: autonomous mobile robots and teleoperated mobile robots [1]. In the field of autonomous mobile robots, relying on a single sensor is no longer sufficient to meet the accuracy and robustness requirements of localization and mapping systems. The integration of multiple sensors and the fusion of their data are core technologies for achieving high-precision positioning and navigation in autonomous robots [2]. Among the sensors commonly used in outdoor scenarios, the GPS global navigation satellite system (GPS GNSS) and GNSS/INS (Inertial Navigation System) are widely utilized. Generally, GPS can provide centimeter-level positioning. However, due to the presence of urban high-rise buildings obstructing the surrounding area, GPS signals often result in inaccurate positioning [3]. Consequently, the upper computer may provide code differences or single-point positioning instead of fixed solutions. Therefore, it is common practice to either resort to teleoperation for remote control [4] or incorporate various types of sensors such as IMU, LiDAR, and cameras to assist with positioning.

Recently, numerous solutions have been proposed to address the localization problem in autonomous mobile robots and unmanned vehicles. One of the most widely adopted approaches is the Extended Kalman Filter [5], which combines GPS and IMU data fusion to estimate the localization position. However, this solution heavily relies on GPS data, and the unavailability of GPS signals can introduce significant errors in the localization results.

To overcome this challenge, computer vision and LiDAR based Simultaneous Localization and Mapping techniques are extensively utilized for state estimation in mobile robots. Compared to cameras, LiDAR can directly capture 3D structural information within a specific radius and is less sensitive to lighting conditions. Therefore, laser-based SLAM enhances system robustness and localization accuracy compared to vision-based SLAM. However, due to its dependence on the surrounding environment, LiDAR can experience drift during fast motion, leading to loss of robot positioning. Researchers addressing this issue commonly employ filtering and graph optimization methods to fuse GPS, LiDAR, and IMU data.

Gao [6] employed an Extended Kalman Filter (EKF) to estimate the positional attitude by loosely coupling GPS and LiDAR, periodically correcting the IMU. Shamsudin [7] utilized a Rao-Blackwellized Particle Filter (RBPF) to integrate GPS and LiDAR data for detecting consistency in petrochemical enterprise maps and constructing maps using GPS and LiDAR data in Fast-SLAM. Abdelaziz [8] implemented SLAM based on a loosely coupled EKF, INS, and LiDAR, matching relative poses of 3D probability maps. The proposed method was tested on the KITTI dataset to validate its robustness. Aboutaleb [9] utilized the EKF with LiDAR data, GNSS, and simplified 3D inertial sensors, employing LO to constrain system positioning drift. LIOM [10] utilized a CNN segmentation network to remove dynamic objects, fusing LiDAR and IMU data using ESKF, and generating a static global map through scan matching. LINS [11] was the first method to employ the Iterated Error State Kalman Filter (IESKF) for tightly-coupled LiDAR and IMU-based robot motion estimation. It recursively corrected the robot’s estimated pose using LiDAR-extracted features, preventing filter divergence during long-term operation while maintaining computational speed. FAST-LIO [12] fused LiDAR point cloud features with IMU data using IESKF, and proposed a dimension-dependent Kalman gain formulation to reduce computational intensity resulting from laser point cloud features. FAST-LIO2 [13] also utilized IESKF and improved positioning by incorporating the original LiDAR point cloud information into the map, enhancing accuracy by observing subtle environmental features. The system employed an incremental K-D tree (IKD tree) data structure for incremental updates and dynamic point cloud smoothing, reducing computational requirements and increasing real-time performance. Li [14] proposed a bilateral haptic teleoperation method to enhance robot adaptability. Faster-LIO [15] replaced the IKD tree with an incremental voxel-based point cloud data structure (iVox) that supports incremental insertion and parallel approximation queries, resulting in an efficient and robust LiDAR inertial odometry (LIO) framework.

Another approach to address the localization problem is to utilize graph optimization [16] in the framework of SLAM algorithms. Although the aforementioned filter-based approach offers significant advantages in terms of localization speed, it falls short of the localization accuracy achieved by graph optimization.

Kukko [17] collected 3D point cloud data from the surrounding forest environment using mobile laser scanning and combined the results with GNSS/INS to optimize the trajectory using a graph optimization method. This approach accurately extracted the forest map and tree parameters. Hess [18] implemented 2D LiDAR localization and mapping based on graph optimization, employing the branch-and-bound method for scan calculation. Chang [19] proposed IMU and ODO pre-integration that incorporates odometry. They used a 3D probabilistic map at the front end to enhance point cloud matching effectiveness in feature-limited environments and ensure system trajectory accuracy when GNSS signals are unavailable. Pierzchała [20] employed a graph-based SLAM system with a 16-line laser rangefinder, camera, IMU, and GPS to evaluate relative distances between wood structures and trees. Google Cartographer [21] achieved the fusion of GNSS, 3D-LiDAR, and IMU data based on graph optimization. This method assumed that the mobile robot moved at a low, uniform speed and used gravity to solve horizontal attitude, neglecting modeling of IMU deviation errors. LOAM [22], proposed by Zhang and Singh, extracted edge and planar features from LiDAR point clouds to reduce computational effort required for matching. The framework used a high-frequency LiDAR odometry for positional estimation at the front end and low-frequency map optimization at the back end for map-building. This resulted in a low-computation, low-drift, and real-time SLAM system. LOAM consistently performed well in the KITTI [23] dataset. Subsequently, several variants and updated versions of LOAM were proposed, including LeGO-LOAM [24], R-LOAM [25], and F-LOAM [26]. These SLAM algorithms primarily focused on improving the processing time of the LOAM algorithm. For in-vehicle navigation systems, the integration of LiDAR SLAM with GNSS/INS plays a crucial role in achieving system redundancy and robustness. Shan’s proposed LIO-SAM [27] utilized point cloud feature extraction with key frames at the front end to reduce computational effort. It also incorporated IMU pre-integration factor, GPS factor, and laser odometry factor into the back-end factor map optimization to construct P^3^-LOAM [28]. The system combines LiDAR-SLAM with GNSS precise point positioning and estimates the covariance of laser SLAM based on the error propagation model of the SVD Jacobi matrix. Additionally, it relies on laser SLAM when the GNSS observations contain significant errors, eliminating PPP outliers and achieving high-accuracy positioning in urban canyon environments.

In summary, filter-based methods primarily rely on the first-order Markov assumption, where the current state depends only on the previous state. While this reduces computational complexity, it also leads to accumulated drift errors. On the other hand, graph optimization-based methods store the states of all previous time steps using keyframes and continuously correct accumulated errors with subsequent observations. However, the drawback is that it requires more computational resources and memory usage.

To address these challenges, this paper proposes a SLAM system that combines filtering and graph optimization, aiming to improve localization speed while maintaining accuracy. The main contributions of this work are as follows:

1. We employ the IESKF algorithm to achieve tight coupling of laser rangefinder and IMU data, enabling robust pose estimation. This approach effectively integrates sensor data to improve the accuracy and stability of frontend pose estimation.
2. We introduce a method for filtering out anomalous GPS data based on GPS state variables and confidence. This method effectively reduces the interference and errors caused by GPS data, thereby enhancing the localization performance of the SLAM system under GPS interruption.
3. We utilize factor graph optimization to fuse the frontend-generated odometry, IMU, GPS, and loop closure detection modules. By constructing a factor graph, the system can leverage the information from each module during the optimization process, improving the overall localization accuracy and consistency of the SLAM system.
4. The proposed SLAM framework’s localization accuracy improvements are tested and evaluated using GPS uninterrupted/interrupted tests on the KITTI dataset and our own experimental platform.

## 2. SLAM System

**Figure 1:**
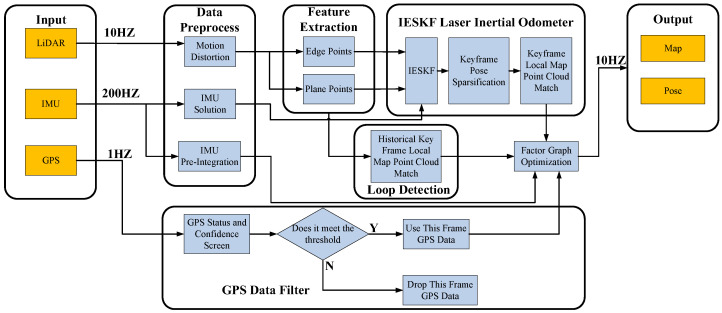
SLAM system framework diagram.

**Figure 2:**
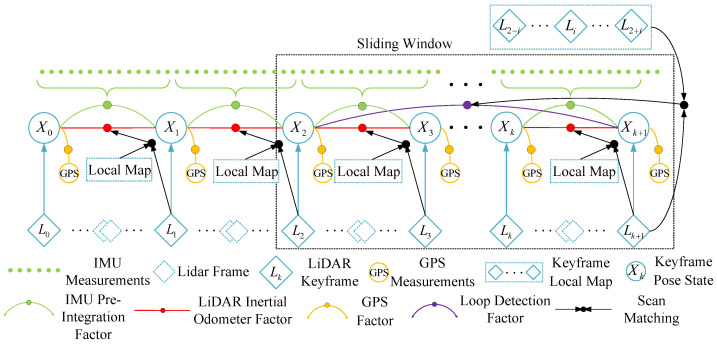
Factor graph model graph.

The framework for the GPS, IMU, and LiDAR localization and mapping system presented in this paper is illustrated in Figure 1. The system comprises several modules, including data preprocessing, front-end IESKF odometry, back-end factor graph global pose optimization, GPS data filtering, and loop closure detection. The front-end utilizes a tightly coupled laser inertial odometry based on IESKF, while the back-end employs a factor graph (shown in Figure 2) to fuse the front-end LIO factors, IMU predicted measurement factors, and GPS factors.

Factor Graph Optimization is a graph model used for probabilistic inference and parameter estimation problems. It employs a graph structure composed of nodes and edges to represent the dependencies and constraints between variables. In this model, nodes represent the variables to be optimized, while edges represent the constraints. By constructing such a graph, we can gain a better understanding of the problem’s structure and utilize optimization algorithms to find the optimal variable configuration. Factor Graph Optimization provides a flexible and effective approach for addressing complex inference and estimation problems.

The process of Factor Graph Optimization involves iteratively minimizing an objective function that measures the difference between the predicted state vector and the actual state vector. In this paper, we employ Incremental Smoothing and Mapping (iSAM2) [29] using Bayesian tree mapping for the factor graph optimization process. The objective of this process is to obtain the posterior pose distribution of the robot, given the known sensor measurement noise, and further model it by iteratively decomposing a set of factors $\phi \left(X\right)$. The objective function is described by Equation (1):

(1) $$\underset{X}{\arg\min}f(X)=\underset{X}{\arg\max}\prod_{i}\phi_{i}\left(X_{i}\right)$$

where X is the vector to be estimated, and f(X) is the cost function.

Equation (1) can be equivalently expressed as a least squares form, as shown in Equation (2):

(2) $$\underset{X}{\arg\min}f(X)≜\underset{X}{\arg\min}\sum_{i}{\left\|h_{i}\left(X_{i}\right)-z_{i}\right\|}_{D_{i}}^{2}$$

where $h\left(X\right)$ is the observation equation, z is the observed value, and D is the covariance matrix of the observed value.

The modeling of the robot state vector x consists of the rotation matrix R∈SO(3), position $p\in {\mathbb{R}}^{3}$, velocity v, and IMU bias b. A transformation T∈SE(3) from the robot base O to the world frame W is represented as T=[R∣p].

(3) $$x={\left[R^{T},p^{T},v^{T},b^{T}\right]}^{T}$$

### 2.1. IESKF LiDAR Inertial Odometry Factor

In this paper, we employ an IESKF in the front-end to achieve tight coupling of sensor data from laser odometry and IMU, aiming to achieve higher algorithm accuracy than LIO-SAM when GPS is interrupted. Compared to traditional Kalman filter methods, this approach offers three main advantages [30]. Firstly, it reduces computational complexity by ignoring second-order products, resulting in a smaller error state. Secondly, it addresses parameterization and gimbal lock problems by keeping the orientation error state small. Thirdly, the slow change in the error state allows for error correction at a lower rate than prediction. The IESKF filtering method used in this paper is based on the approaches presented in LINS and FAST-LIO, and the algorithm steps are outlined below:

(1) Input the posterior state variables ${\overset{⌢}{x}}_{k}$ and covariance matrix ${\overset{⌢}{P}}_{k}$ output by the previous IESKF, the laser point cloud after motion compensation, and the IMU data collected during the current laser scan.
(2) Predict the state variables and covariance matrix as shown in Equations (4) and (5). In Equation (4): ⊕ denotes the generalized addition; ${\overset{⌣}{x}}_{i+1}$ and ${\overset{⌣}{x}}_{k}$ respectively represent the prior system state variables between the laser frames k-th and k+1-th when receiving IMU data at times i and i+1; $T=t_{i+1}-t_{i}$ denotes the IMU sampling period; $f({\overset{⌣}{x}}_{i},u_{i},\omega_{i})$ denotes the system state transition matrix; and $u_{i}$ and $\omega_{i}$ represent the IMU measurement values and their measurement noise at time i. In Equation (5): ${\overset{⌣}{P}}_{i+1}$ represents the predicted covariance matrix at time i+1; $F_{i}$ represents the predicted state matrix at time i; $B_{i}$ represents the noise matrix; Q represents the noise covariance matrix; and ${\overset{⌢}{P}}_{k}$ represents the posterior covariance matrix of the laser k-th frame.
  (4) $$\begin{array}{cc} {\overset{⌣}{x}}_{i+1} & ={\overset{⌣}{x}}_{i}⊕[T\cdot f({\overset{⌣}{x}}_{i},u_{i},\omega_{i})]\\ {\overset{⌣}{x}}_{0} & ={\overset{⌢}{x}}_{k} \end{array}$$
  (5) $$\begin{array}{cc} {\overset{⌣}{P}}_{i+1} & =F_{i}{\overset{⌣}{P}}_{i}F_{i}^{T}+B_{i}QB_{i}^{T}\\ {\overset{⌣}{P}}_{0} & ={\overset{⌢}{P}}_{k} \end{array}$$
(3) Setting the initial value of the iteration count α to 1, the state quantity of the iteration is ${\overset{⌣}{x}}_{k+1}^{\alpha =0}={\overset{⌣}{x}}_{k+1}$.
(4) Judge whether the absolute value of the difference between the state quantity obtained after one iteration and the previous iteration is less than the threshold ∂, represented by the symbol in Formula (6), where ⊖ denotes generalized subtraction. If it is less than the threshold ∂, then repeat the following loop.
  (6) $$\left\|{\overset{⌣}{x}}_{k+1}^{\alpha +1}⊖{\overset{⌣}{x}}_{k+1}^{\alpha }\right\|<\partial$$
  (a) Calculate the Jacobian matrix $J_{k+1}^{\alpha }$ of the error state vector at $\delta x_{k+1}^{\alpha }=0$ point using Formula (7), where $\delta x_{k+1}$ represents the error state vector of k+1-th frame. Use Formula (8) to update the prior covariance matrix ${\overset{⌣}{P}}_{k+1}$ during the iteration process.
    (7) $$\begin{array}{cc} \delta x_{k+1} & ={\overset{⌣}{x}}^{\alpha }{}_{k+1}⊖{\overset{⌣}{x}}_{k+1}+J_{k+1}^{\alpha }\delta x_{k+1}^{\alpha }\\ J_{k+1}^{\alpha } & =\left[\begin{array}{cc} A_{k+1}{\left(\delta \theta_{k+1}\right)}^{-T} & 0_{3\times 15}\\ 0_{15\times 3} & I_{15\times 15} \end{array}\right] \end{array}$$
    (8) $${\overset{⌣}{P}}_{k+1}={\left(J_{k+1}^{\alpha }\right)}^{-1}{\overset{⌣}{P}}_{k+1}{\left(J_{k+1}^{\alpha }\right)}^{-T}$$
  (b) Transform the laser point cloud into the world coordinate system, and calculate the residual equation $f(x_{k+1}^{k})$ and covariance matrix $H_{k+1}$ of the observation using Formulas (9) and (10), respectively. Here, ${\tilde{X}}^{L_{e}}{}_{\left(k+1,i\right)}$ and ${\tilde{X}}^{L_{s}}{}_{\left(k+1,i\right)}$ represent the coordinate sets of feature points after motion compensation for corner points $L_{e}$ and plane points $L_{s}$, respectively, between k-th frame and k+1-th frame. The covariance matrix $H_{k+1}$ is represented using the formula from LINS, $R_{k+1}^{k}$ represents the pose transformation of the laser between k-th frame and k+1-th frame, and ${\left[•\right]}_{\times }$ denotes the skew-symmetric matrix of the variable.
    (9) $$f(x_{k+1}^{k})=\left\{\begin{array}{l} \frac{\left|\left({\tilde{X}}^{L_{e}}{}_{\left(k+1,i\right)}-X_{\left(k+1,i\right)}^{L_{e}}\right)\times \left({\tilde{X}}^{L_{e}}{}_{\left(k+1,i\right)}-X_{\left(k,l\right)}^{L_{e}}\right)\right|}{\left|X_{\left(k,j\right)}^{L_{e}}-X_{\left(k,l\right)}^{L_{e}}\right|}\\ \frac{\left|({\tilde{X}}^{L_{s}}{}_{\left(k+1,i\right)}-X_{\left(k,j\right)}^{L_{s}})\left(\left(X_{\left(k,j\right)}^{L_{s}}-X_{\left(k,l\right)}^{L_{s}})\times (X_{\left(k,j\right)}^{L_{s}}-X_{\left(k,m\right)}^{L_{s}}\right)\right)\right|}{\left|\left(X_{\left(k,j\right)}^{L_{s}}-X_{\left(k,l\right)}^{L_{s}}\right)\times \left(X_{\left(k,j\right)}^{L_{s}}-X_{\left(k,m\right)}^{L_{s}}\right)\right|} \end{array}\right.$$
    (10) $$\begin{array}{cc} H_{k+1} & =\frac{\partial f}{\partial {\tilde{X}}_{\left(k+1,i\right)}^{L}}\cdot \frac{\partial {\tilde{X}}_{\left(k+1,i\right)}^{L}}{\partial \delta x}\\ & =\left\{\begin{array}{c} \left\{\frac{\left\{{\left[\left({\tilde{X}}_{\left(k+1,i\right)}^{L_{e}}-X_{\left(k+1,i\right)}^{L_{e}}\right)\times \left({\tilde{X}}_{\left(k+1,i\right)}^{L_{e}}-X_{\left(k,l\right)}^{L_{s}}\right)\right]}^{T}\right.}{\left|\left({\tilde{X}}_{\left(k+1,i\right)}^{L_{e}}-X_{\left(k+1,i\right)}^{L_{e}}\right)\times \left({\tilde{X}}_{\left(k+1,i\right)}^{L_{e}}-X_{\left(k,l\right)}^{L_{s}}\right)\right|}\right\}\cdot \left[R_{k+1}^{k}{\left(X_{\left(k+1,i\right)}^{L_{s}}\right)}_{\times },I\right]\\ \left\{\frac{{\left[\left(X_{\left(k,j\right)}^{L_{s}}-X_{\left(k,l\right)}^{L_{s}}\right)\times \left(X_{\left(k,j\right)}^{L_{s}}-X_{\left(k,m\right)}^{L_{s}}\right)\right]}^{T}}{\left|\left(X_{\left(k,j\right)}^{L_{s}}-X_{\left(k,l\right)}^{L_{s}}\right)\times \left(X_{\left(k,j\right)}^{L_{s}}-X_{\left(k,m\right)}^{L_{s}}\right)\right|}\right\}\cdot \left[R_{k+1}^{k}{\left(X_{\left(k+1,i\right)}^{L_{s}}\right)}_{\times },I\right] \end{array}\right. \end{array}$$
  (c) Update the state variables ${\overset{⌣}{x}}_{k+1}^{\alpha +1}$ and Kalman gain $K_{k+1}$ using Formulas (11) and (12).
    (11) $${\overset{⌣}{x}}_{k+1}^{\alpha +1}={\overset{⌣}{x}}_{k+1}^{\alpha }-K_{k+1}f_{k+1}^{\alpha }-(I-K_{k+1}H_{k+1}){\left(J_{k+1}^{\alpha }\right)}^{-1}\left({\overset{⌣}{x}}_{k+1}^{\alpha }⊖{\overset{⌣}{x}}_{k+1}\right)$$
    (12) $$K_{k+1}={\left({\overset{⌣}{P}}^{-1}{}_{k+1}+H_{k+1}^{T}L_{k+1}^{-1}H_{k+1}\right)}^{-1}H_{k+1}^{T}L_{k+1}^{-1}$$
(5) Output the posterior state quantity ${\overset{⌢}{x}}_{k+1}$ and posterior covariance ${\overset{⌢}{P}}_{k+1}$ using Equations (13) and (14).
  (13) $${\overset{⌢}{x}}_{k+1}={\overset{⌣}{x}}^{\alpha +1}{}_{k}$$
  (14) $${\overset{⌢}{P}}_{k+1}=(I-K_{k+1}H_{k+1}){\overset{⌣}{P}}_{k+1}$$

During factor graph optimization, there is redundant information between IMU data and laser point cloud data. Including IESKF odometry data of each frame in the back-end factor graph optimization only leads to a slight improvement in localization accuracy but consumes a significant amount of computational resources, which ultimately affects the localization accuracy of the system. Therefore, we employ the keyframe and sliding window strategy in factor graph optimization to reduce the computational resources required in the back-end. Keyframes are selected based on representative laser-IMU odometry data over a specific period, helping to reduce the data volume. In the sliding window approach, only the keyframe data within the window is optimized, while the regular data frames are discarded.

In this paper, “ordinary frames” refers to laser frames observed by IESKF, while “keyframes” are determined based on position or attitude changes estimated by IESKF’s laser inertial odometry exceeding 1 m or 5°, respectively. Adjacent keyframes are utilized to construct local maps. Based on the current keyframe pose, *i* nearest keyframes are extracted to form the adjacent keyframe set $\left\{F_{i-k},\cdots ,F_{k}\right\}$, and the poses corresponding to the adjacent keyframe set are transformed to the current keyframe F coordinate system. After the transformation, the adjacent keyframe point clouds are merged into one local map. As subsequent new keyframe point clouds are added to the local map, keyframe point clouds that are far away from the local map are removed. To obtain a more accurate pose transformation relationship between two keyframes, this paper adopts the ICP registration algorithm to match the current keyframe with the local map and derive the pose transformation relationship. The residual equation between the *k*-th and *k*+1-th laser radar keyframes can be obtained as shown in Equation (15):

(15) $$r_{L}=\left[\begin{array}{c} \Delta t-R_{k}^{T}\left(t_{k+1}-t_{k}\right)\\ \log\left(\Delta R^{T}R_{k}^{T}R_{k+1}\right) \end{array}\right]$$

### 2.2. GPS Factor

Due to significant fluctuations in GPS data in highly urbanized environments, this paper incorporates a GPS state and confidence filtering approach to screen the available GPS data and include them as GPS factors in the factor graph. This method aims to exclude anomalous GPS data from being included in the factor graph, thereby ensuring higher accuracy. Specifically, this paper only utilizes GPS data with fixed solutions and narrow lane fixed solutions.

In geometric positioning methods, the accuracy of localization is influenced by the relative distances between multiple base stations and mobile stations, which is commonly referred to as Dilution of Precision (DOP). To calculate the DOP factor, we introduce the GPS single-point positioning model, which is represented by the following equation:

(16) $$\rho_{j}=\sqrt{{\left(x_{j}-x_{u}\right)}^{2}+{\left(y_{j}-y_{u}\right)}^{2}+{\left(z_{j}-z_{u}\right)}^{2}}+c\left(t_{u}-t_{j}\right)$$

where $\left(x_{u},y_{u},z_{u}\right)$ is the receiver coordinates, $\left(x_{j},y_{j},z_{j}\right)$ is the base station coordinates, $t_{u}$ and $t_{j}$ are the clock bias between the receiver and the base station, $\rho_{j}$ represents the pseudorange from the receiver to the base station, and j represents the number of visible base stations.

Given the approximate values of receiver coordinates $\left(\tilde{x_{u}},\tilde{y_{u}},\tilde{z_{u}}\right)$, and clock error $\tilde{t_{u}}$, we can linearize the positioning model by performing a first-order Taylor series expansion, as shown in Equation (17). Furthermore, we can represent Equation (17) in matrix form as Equation (18):

(17) $$\Delta \rho_{j}=l_{j}\Delta x_{u}+m_{j}\Delta y_{u}+n_{j}\Delta z_{u}-c\Delta t_{u}$$

(18) ΔP=HΔX

(19) $$\Delta P=\left[\begin{array}{c} \Delta \rho_{1}\\ \Delta \rho_{2}\\ \vdots \\ \Delta \rho_{j} \end{array}\right],\;H=\left[\begin{array}{cccc} l_{1} & m_{1} & n_{1} & 1\\ l_{2} & m_{2} & n_{2} & 1\\ \vdots & \vdots & \vdots & \vdots \\ l_{j} & m_{j} & n_{j} & 1 \end{array}\right],\;\Delta X=\left[\begin{array}{c} \Delta x_{u}\\ \Delta y_{u}\\ \Delta z_{u}\\ -c\Delta t_{u} \end{array}\right]$$

where $l_{j}$, $m_{j}$, and $n_{j}$ represent the direction cosines of the unit vector pointing from the approximate position towards the j-th base station.

By applying the least squares method to solve Equation (18), we can derive quantitative expressions for the components of the symmetric matrix G, which represent the accuracy factors. These expressions are given by the following equation:

(20) $$G={\left(H^{T}H\right)}^{-1}=\left[\begin{array}{llll} g_{11} & g_{12} & g_{13} & g_{14}\\ g_{12} & g_{22} & g_{23} & g_{24}\\ g_{13} & g_{23} & g_{33} & g_{34}\\ g_{14} & g_{24} & g_{34} & g_{44} \end{array}\right]$$

We select the square root of the sum of squared errors in dimensions, precision, and elevation as the confidence criterion, which is commonly known as PDOP (Position Dilution of Precision). It can be calculated using the following equation:

(21) $$PDOP=\sqrt{g_{11}+g_{22}+g_{33}}$$

**Figure 3:**
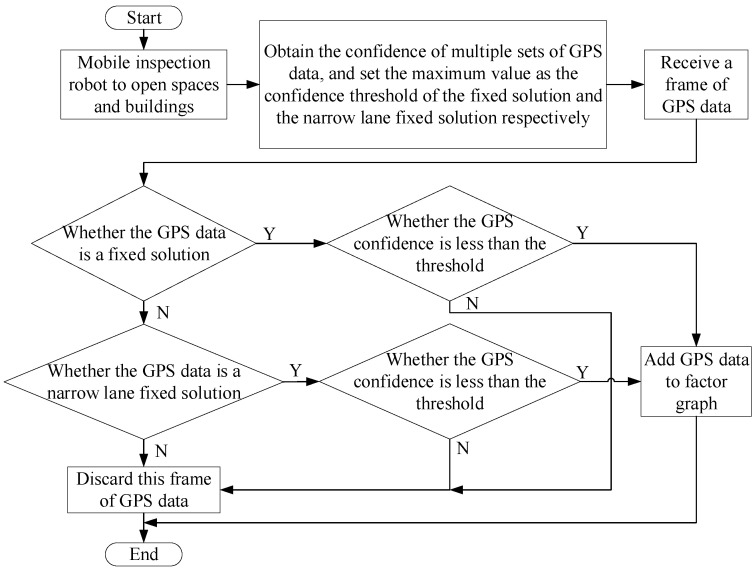
Flow chart of screening strategies based on GPS status and confidence.

First, the inspection robot is moved to an open area to allow for movement, and multiple confidence values are recorded when the GPS state is a fixed solution. The maximum value among these recorded confidences is selected as the threshold for fixed solution confidence. Second, the steps mentioned above are repeated to obtain the confidence threshold for narrow lane fixed solutions. Finally, during the operation of the SLAM system, the corresponding confidence threshold is selected based on the GPS state. This threshold is then compared with the current confidence value, and GPS data with a confidence lower than the threshold are considered usable. Figure 3 illustrates the flowchart of the GPS state and confidence filtering strategy employed in this paper.

After filtering out unreliable data based on confidence, we utilize the remaining GPS data to calculate the coordinates in the W-frame for latitude, longitude, and altitude. These coordinates are then incorporated into the factor graph as a GPS position constraint cost function. The GPS position constraint cost function is represented by the following equation:

(22) $$\sum_{i\in G}{\left\|e\left(R_{t_{i}}^{o},R_{o}^{w}\right)\right\|}_{D_{g}}^{2}=\sum_{i\in G}{\left\|R_{o}^{w}\left(R_{t_{i}}^{o}T_{g}^{b}\right)-T_{g}^{w}\right\|}_{D_{g}}^{2}$$

where $R_{t_{i}}^{o}$ is the pose of the IMU in the vehicle coordinate system at time $t_{i}$, $R_{o}^{w}$ is the transformation parameters between the vehicle coordinate system and the global coordinate system W, $T_{g}^{w}$ is the GNSS positioning result in the global coordinate system W, $T_{g}^{b}$ is the antenna lever arm for GNSS, $D_{g}$ is the variance-covariance matrix for $T_{g}^{w}$ provided by the GNSS RTK positioning solution, and G is the set of nodes with GNSS position correction.

**Figure 4:**
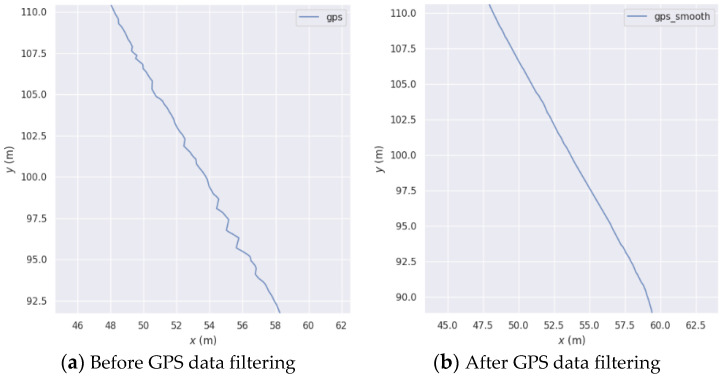
Filter GPS comparison chart based on GPS status and confidence.

We conducted tests in practical scenarios, specifically at the rear of the supporting service center building in the Intelligent Technology Park, where GPS data exhibited fluctuations. Figure 4 illustrates the GPS trajectories before and after applying our filtering approach. It can be observed that the GPS data exhibits significant fluctuations prior to filtering, whereas the fluctuations are reduced after the application of our filtering method.

### 2.3. Loop Detection Factor

Similar to LIO-SAM, this paper employs a keyframe-based Euclidean distance approach for loop closure detection. Firstly, the laser point cloud is transformed into the world coordinate system. Based on the position of the current keyframe, a search range of distance d is defined to identify historical keyframes that are in close proximity and have a longer detection time. The positions of the keyframes within the search range are further filtered based on a specified time interval. The local feature point cloud map is constructed by aggregating feature point clouds from a range of 25 frames centered around the identified historical keyframes. The current keyframe is then matched with the local feature point cloud map using ICP point cloud registration to determine the relative pose transformation relationship in the world coordinate system. For a more comprehensive explanation of the loop closure detection process, please refer to LIO-SAM.

### 2.4. IMU Pre-Integration

The angular velocity and acceleration measured from the IMU are defined as follows:

(23) $${\overset{⌢}{\omega }}_{t}=\omega_{t}+b_{t}^{\omega }+n_{t}^{\omega }$$

(24) $${\overset{⌢}{a}}_{t}=R_{t}^{BW}\left(a_{t}-g\right)+b_{t}^{a}+n_{t}^{a}$$

where ${\overset{⌢}{\omega }}_{t}$ and ${\overset{⌢}{a}}_{t}$ are the measurements of the IMU at the moment *t* and the *B* coordinate systems, ${\overset{⌢}{\omega }}_{t}$ and ${\overset{⌢}{a}}_{t}$ are subject to the slowly transformed bias $b_{t}$ and white noise $n_{t}$, $R_{t}^{BW}$ is the rotation matrix from the coordinate system *W* to *B,* and *g* is a fixed gravity vector in the *W* coordinate system.

The robot motion was then inferred from the IMU measurements. The position, attitude, and velocity of the robot during t+Δt were calculated as follows:

(25) $$v_{t+\Delta t}=v_{t}+g\Delta t+R_{t}({\overset{⌢}{a}}_{t}-b_{t}^{a}-n_{t}^{a})\Delta t$$

(26) $$p_{t+\Delta t}=p_{t}+v_{t}\Delta t+\frac{1}{2}g\Delta t^{2}+\frac{1}{2}R_{t}\left({\overset{⌢}{a}}_{t}-b_{t}^{a}-n_{t}^{a}\right)\Delta t^{2}$$

(27) $$R_{t+\Delta t}=R_{t}\exp(({\overset{⌢}{\omega }}_{t}-b_{t}^{\omega }-n_{t}^{\omega })\Delta t)$$

where $R=R_{t}^{WB}=R_{t}^{BW}{}^{T}$$R_{t}^{BW}$ are the rotation matrices of the coordinate systems *B* to *W*, and the angular velocity and acceleration of the base coordinates *B* are assumed to be constant during the integration process. Then, we use the IMU pre-integration method to obtain the relative motion of the carriers within adjacent timestamps, where the pre-integrated measurements $\Delta v_{ij}$, $\Delta p_{ij}$, and $\Delta R_{ij}$ between moments *i* and *j* are calculated by the following equations:

(28) $$\Delta v_{ij}=R_{i}{}^{T}(v_{j}-v_{i}-g\Delta t_{ij})$$

(29) $$\Delta p_{ij}=R_{i}{}^{T}(p_{j}-p_{i}-v_{i}\Delta t_{ij}-\frac{1}{2}g\Delta t_{ij}{}^{2})$$

(30) $$\Delta R_{ij}=R_{i}{}^{T}R_{j}$$

## 3. Experimental

### 3.1. KITTI Dataset Testing and Evaluation

This paper presents an experimental study on the fusion of filtering and graph optimization SLAM algorithms using the KITTI datasets 05, 07, and 10. The trajectories and ATE statistical indicators generated are compared and analyzed with an open-source multi-sensor fusion SLAM algorithm using the EVO [31] trajectory evaluation tool. The filtering algorithm used in this experiment is FAST-LIO, while the graph optimization algorithm used is LIO-SAM. To evaluate the robustness of the algorithm when GPS is suddenly interrupted, GPS data is also interrupted. Since FAST-LIO does not incorporate GPS, its accuracy is not affected by GPS interruption. Therefore, its ATE statistical indicator is not presented in the GPS interruption experiment.

#### 3.1.1. GPS Uninterrupted Experiment

**Figure 5:**
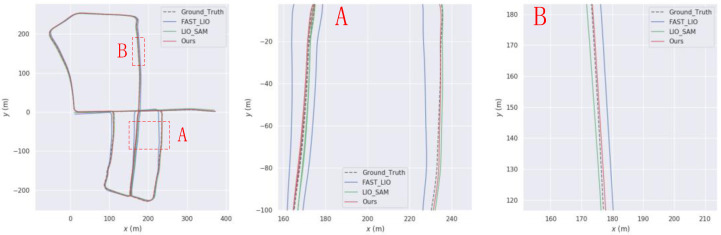
Comparison of our trajectory with LIO-SAM and FAST-LIO on the KITTI05 sequence. (**A**) and (**B**) are the sections where our trajectory is better than LIO-SAM and FAST-LIO.

**Figure 6:**
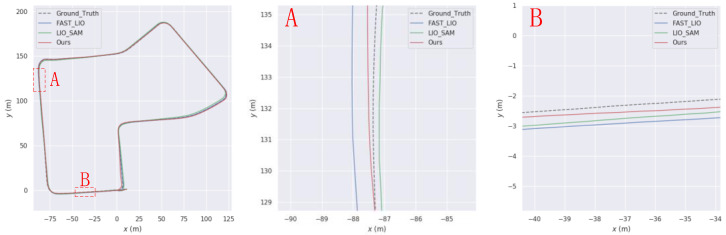
Comparison of our trajectory with LIO-SAM and FAST-LIO on the KITTI07 sequence. (**A**) and (**B**) are the sections where our trajectory is better than LIO-SAM and FAST-LIO.

**Figure 7:**
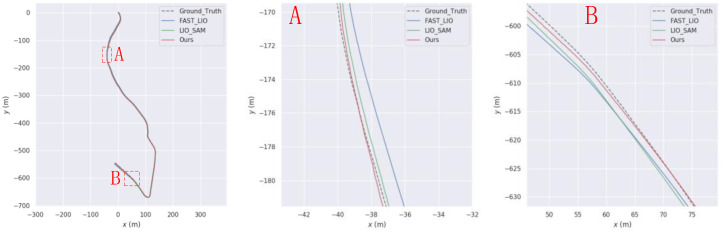
Comparison of our trajectory with LIO-SAM and FAST-LIO on the KITTI10 sequence. (**A**) and (**B**) are the sections where our trajectory is better than LIO-SAM and FAST-LIO.

**Figure 8:**
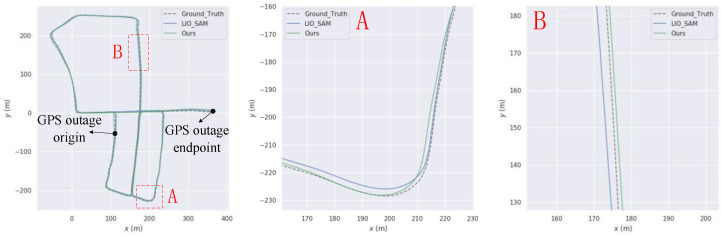
Comparison of our trajectory with LIO-SAM on the KITTI05 sequence. (**A**) and (**B**) are the road sections where our trajectory is better than LIO-SAM.

To start, we performed the GPS non-interruption experiment and obtained trajectories for sequences 05, 07, and 10, as depicted in Figure 5, Figure 6, Figure 7 and Figure 8, respectively. Our algorithm exhibited superior trajectory results compared to the two open-source algorithms, with trajectories that closely aligned with the true values. 

In Table 1, we present our algorithm alongside the ATE statistical data for FAST-LIO and LIO-SAM.

In terms of performance metrics on the 05 sequence, the algorithm proposed in this paper outperformed both LIO-SAM and FAST-LIO. Specifically, it achieved improvements of 21.57%, 44.75%, 43.19%, and 12.22% in maximum error, average error, root mean square error, and minimum error, respectively, when compared to LIO-SAM. Furthermore, compared to FAST-LIO, the proposed algorithm showed improvements of 74.09%, 79.64%, 63.68%, and 58.57% in the same metrics.

On the 07 sequence, the algorithm presented in this paper did not perform as well as LIO-SAM in terms of minimum error. However, it outperformed both LIO-SAM and FAST-LIO in other statistical indicators. Specifically, it achieved improvements of 20.14% and 52.19% in maximum error, 30.95% and 61.28% in average error, and 43.47% and 61.27% in root mean square error, respectively.

Finally, on the 10 sequence, the algorithm proposed in this paper outperformed both LIO-SAM and FAST-LIO in all metrics. In particular, it achieved improvements of 32.42% and 50.24% in maximum error, 48.09% and 58% in average error, 46.28% and 57.9% in root mean square error, and 65.88% and 47.13% in minimum error, respectively.

#### 3.1.2. GPS Interrupted Experiment

**Figure 9:**
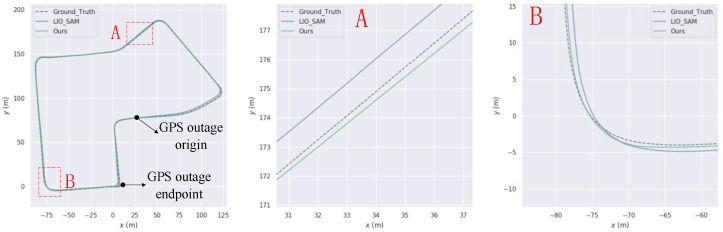
Comparison of our trajectory with LIO-SAM on the KITTI07 sequence. (**A**) and (**B**) are the road sections where our trajectory is better than LIO-SAM.

**Figure 10:**
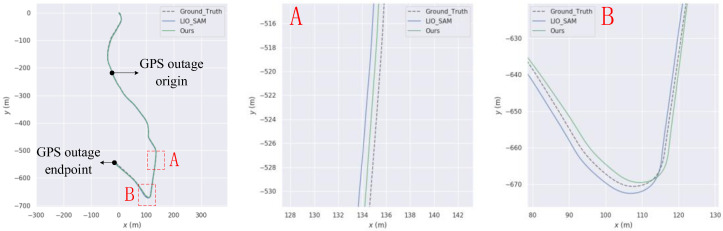
Comparison of our trajectory with LIO-SAM on the KITTI10 sequence. (**A**) and (**B**) are the road sections where our trajectory is better than LIO-SAM.

This paper aims to simulate sudden interruptions of GPS signals by applying interruption processing to GPS data in the KITTI dataset, starting from the 20th second. Specifically, interruptions of approximately 268 s, 95 s, and 107 s were applied to the KITTI05, KITTI07, and KITTI10 sequences, respectively. The trajectories of these sequences are shown in Figure 8, Figure 9 and Figure 10, respectively. From Figures A and B in each of these figures, it can be observed that the trajectory generated by the algorithm proposed in this paper is superior to that generated by LIO-SAM in GPS signal interruption scenarios.

Table 2 presents the ATE statistical data for the proposed algorithm and LIO-SAM in GPS signal interruption scenarios.

For the KITTI05 sequence, the proposed algorithm has a slightly higher minimum error value than LIO-SAM, with an increase of 0.126808 m. However, the proposed algorithm outperforms LIO-SAM in all other statistical indicators, with improvements of 40.92%, 29.01%, and 28.2%, respectively.

For the KITTI07 sequence, the proposed algorithm has a slightly higher minimum error value than LIO-SAM, but it outperforms LIO-SAM in all other statistical indicators, with improvements of 43.13%, 13.85%, and 17.06%, respectively.

For the KITTI10 sequence, the proposed algorithm has a slightly higher minimum error value than LIO-SAM, with an increase of 0.209469 m. However, the proposed algorithm outperforms LIO-SAM in all other indicators, with improvements of 50.96%, 21.14%, and 29.75%, respectively.

### 3.2. Experiments on a Real Platform

**Figure 11:**
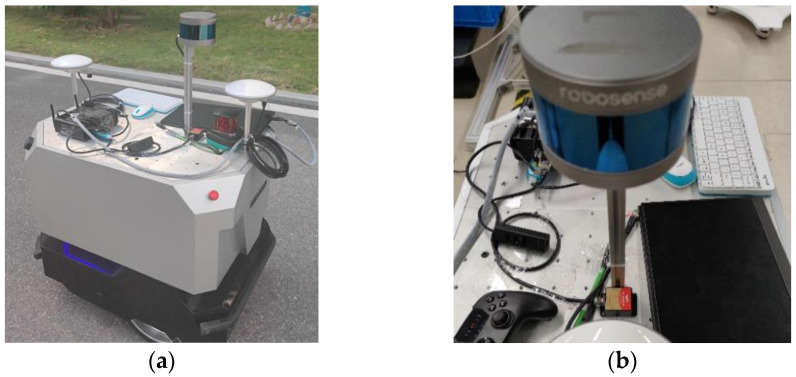
(**a**) Outdoor autonomous mobile robot experimental platform equipped with GPS, LiDAR, and IMU sensors for data collection; and (**b**) closer view of the LiDAR and IMU sensors.

In a subsequent evaluation of the method proposed in this paper, we conducted localization map-building experiments on an actual autonomous mobile robot in an outdoor environment. All datasets of real scenes in the park were collected on an experimental platform, as shown in Figure 11. The experimental platform was equipped with a 1.60 GHz Intel i5-8250U IPC and connected to an RS-LiDAR-16 LiDAR with a frequency of 10 Hz, a Witt Smart IWT905 IMU with a frequency of 200 Hz, and a GPS consisting of a 10 Hz BeiDou XingTong base station NC502-D and a mobile station NC507-S.

**Figure 12:**
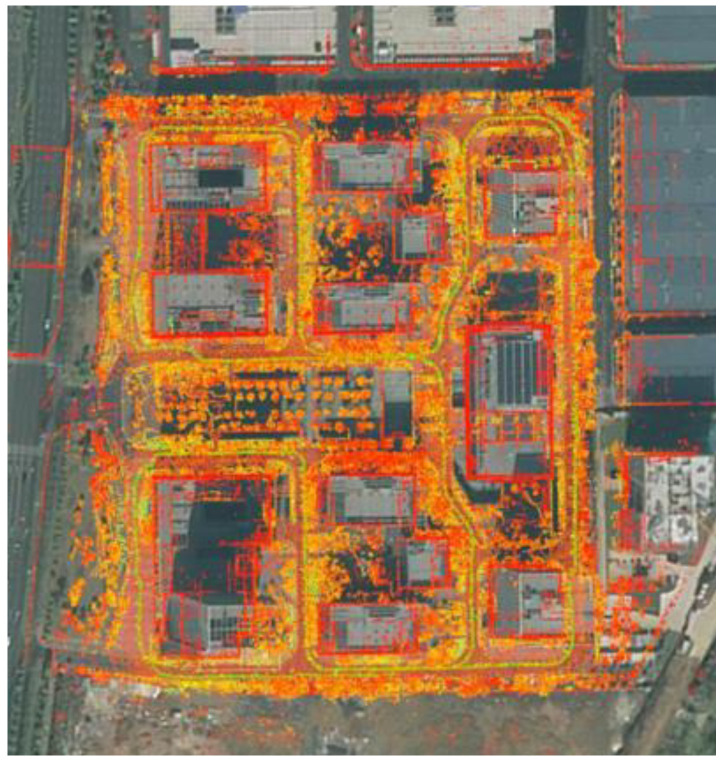
Point cloud map built on our own dataset using our method.

An experiment was conducted to evaluate the positioning accuracy of our algorithm applied to data collected with a mobile autonomous robot in an outdoor environment where fluctuations or interruptions occurred in the GPS data. A navigation area with an approximate size of 280 × 280 m^2^ was created in the park, and the resulting map is presented in Figure 12.

#### 3.2.1. GPS Uninterrupted Experiment

**Figure 13:**
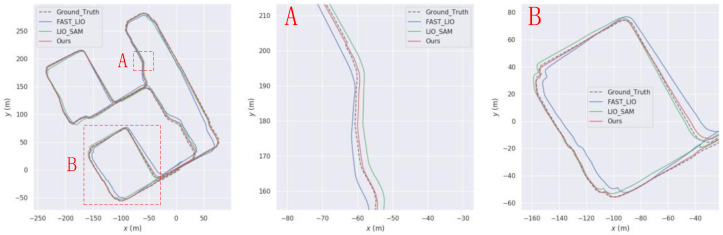
Comparison charts with LIO-SAM trajectory on our own data set (**A**) and (**B**) are road sections where our trajectory is better than LIO-SAM.

First, an experiment was conducted with uninterrupted GPS, and the resulting trajectory within the intelligent technology park is illustrated in Figure 13. The trajectory generated by the algorithm proposed in this paper closely aligns with the true trajectory, demonstrating superior trajectory accuracy compared to LIO-SAM. For instance, in the road section depicted in Figure 13A, the algorithm proposed in this paper exhibits the closest match to the true trajectory. Similarly, in the road section shown in Figure 13B, the proposed algorithm outperforms both LIO-SAM and FAST-LIO.

Within the intelligent technology park, Table 3 presents the ATE statistical data comparing the algorithm proposed in this paper, FAST-LIO, and LIO-SAM. In all statistical indicators, the algorithm proposed in this paper outperforms the two mentioned open-source algorithms. The maximum error value has been improved by 51.03% and 64.64%, the average error value has been improved by 21.68% and 55.5%, the root mean square error has been improved by 24.98% and 58.58%, and the minimum error value has been improved by 71.30% and 68.92%, respectively. These results affirm the high accuracy of the algorithm proposed in this paper.

#### 3.2.2. GPS Interrupted Experiment

**Figure 14:**
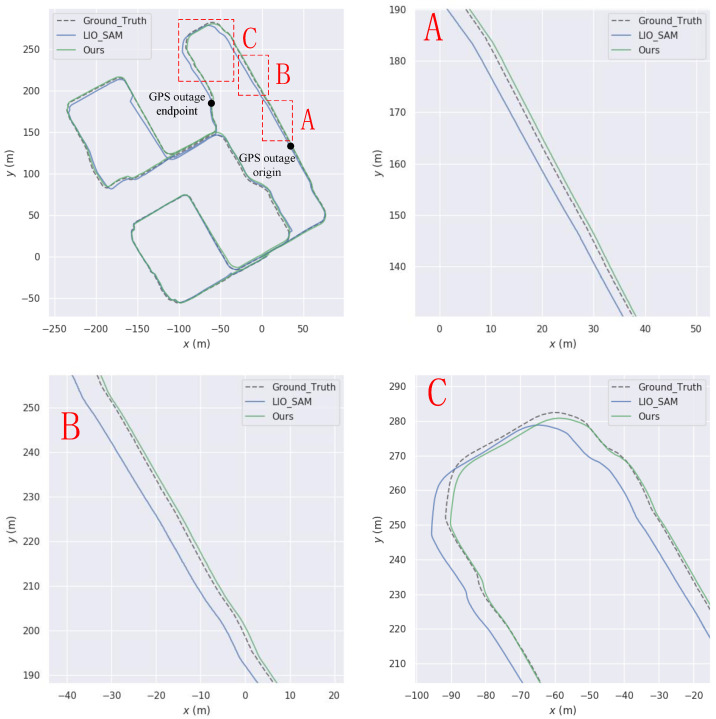
Comparison with LIO-SAM trajectory on our own data set (**A**–**C**) are road sections where our trajectory is better than LIO-SAM.

The experiment was conducted with intermittent GPS signal, and its trajectory within the Intelligent Technology Park is shown in Figure 14. Table 4 shows the ATE statistical data of this paper’s algorithm and LIO-SAM under GPS interruption conditions.

Despite varying GPS interruption times, the minimum error value remains unchanged, and the maximum error value changes relatively little. This is because the poses associated with the minimum and maximum error values in the entire trajectory of the Intelligent Technology Park are not part of the trajectory produced during GPS interruption. Therefore, the subsequent analysis of ATE statistical indicators will exclude the maximum and minimum error values.

Under the GPS interruption condition for 50 s, this paper’s algorithm shows better average error and root-mean-square error compared to LIO-SAM, with improvements of 19.61% and 30.53%, respectively. Under the GPS interruption condition for 100 s, this paper’s algorithm demonstrates improvements of 17.33% and 29.11% in terms of average error and root-mean-square error compared to LIO-SAM. Under the GPS interruption condition for 200 s, this paper’s algorithm still outperforms LIO-SAM, with improvements of 12.05% and 23.14%, respectively. These results indicate that this paper’s algorithm maintains relatively good accuracy improvement even when GPS data is suddenly interrupted for 50 s, and the improvement rate gradually decreases with longer GPS data interruption time. However, the overall trajectory accuracy of this paper’s algorithm remains higher than that of LIO-SAM.

## 4. Conclusions

This paper presents a SLAM system that combines the IESKF and factor graph approaches. The proposed algorithm tightly integrates the laser rangefinder and IMU to obtain an initial pose estimation using IESKF in the front-end. In the back-end, it fuses front-end odometry, IMU, GPS, and loop closure factors, using a factor graph to achieve precise pose estimation. Additionally, a filtering strategy based on GPS status and confidence is applied to remove abnormal GPS data. Multiple experiments were conducted on the KITTI dataset and our own dataset to verify the accuracy of the SLAM system in scenarios with GPS signal interruption. The results demonstrate that our method outperforms LIO-SAM and FAST-LIO in terms of accuracy when GPS data is interrupted.

## Figures and Tables

**Table 1:** Comparing the absolute error of our method, LIO-SAM and FAST-LIO on the KITTI dataset.

Sequence	Method	Max (m)	Mean (m)	Median (m)	Min (m)
05	Ours	1.572958	**0.555240**	**0.623827**	**0.054046**
	LIO-SAM	**1.140164**	0.604368	0.666729	0.050848
	FAST-LIO	12.143285	5.453287	6.046867	0.743559
07	Ours	**1.333466**	**0.555258**	**0.605827**	**0.079949**
	LIO-SAM	**1.323349**	0.617591	0.665901	0.147816
	FAST-LIO	4.248161	2.376953	2.522683	0.456325
10	Ours	**2.751671**	**1.146823**	**1.293698**	0.262802
	LIO-SAM	5.528663	1.544244	1.914991	**0.140108**
	FAST-LIO	5.772416	2.933960	3.189745	0.497090

**Table 2:** Comparing the absolute error of our method with LIO-SAM on KITTI.

Sequence	Method	Max (m)	Mean (m)	Median (m)	Min (m)
05	Ours	**2.936086**	**1.242616**	**1.392347**	0.177911
	LIO-SAM	4.969321	1.750427	1.939206	0.051103
07	Ours	**1.272413**	**0.667964**	**0.723489**	**0.139385**
	LIO-SAM	2.237385	0.775331	0.872355	**0.135207**
10	Ours	**2.978839**	**1.422265**	**1.524391**	0.331625
	LIO-SAM	6.073832	1.803528	2.169900	**0.122156**

**Table 3:** Comparing the absolute error of our method with LIO-SAM on our own dataset.

Method	Max (m)	Mean (m)	Median (m)	Min (m)
Ours	**7.827892**	**3.801397**	**4.189983**	**0.245139**
LIO-SAM	15.985649	4.853936	5.585050	0.854267
FAST-LIO	22.136492	8.540459	10.115659	0.788699

**Table 4:** Comparing the absolute error of our method with LIO-SAM on our own dataset.

Time(s)	Method	Max (m)	Mean (m)	Median (m)	Min (m)
50	Ours	**7.955014**	**4.338442**	**4.700273**	**0.245139**
	LIO-SAM	15.671642	5.396973	6.765650	0.854267
100	Ours	**7.955014**	**4.338442**	**4.700273**	**0.245139**
	LIO-SAM	15.671642	5.396973	6.765650	0.854267
200	Ours	**7.927294**	**4.952018**	**5.397438**	**0.245139**
	LIO-SAM	15.90683	5.630523	7.022866	0.854267

## Data Availability

Not applicable.

## References

[1] Li J., You B., Ding L., Yu X., Li W., Zhang T., Gao H. (2022). Dual-Master/Single-Slave Haptic Teleoperation System for Semiautonomous Bilateral Control of Hexapod Robot Subject to Deformable Rough Terrain. IEEE Trans. Syst. Man Cybern. Syst..

[2] Qin T., Li P., Shen S. (2018). Vins-mono: A robust and versatile monocular visual-inertial state estimator. IEEE Trans. Robots.

[3] Li X., Zhang W., Zhang K., Zhang Q., Li X., Jiang Z., Ren X., Yuan Y. (2021). GPS satellite differential code bias estimation with current eleven low earth orbit satellites. J. Geod..

[4] You B., Li J., Ding L., Xu J., Li W., Li K., Gao H. (2018). Semi-Autonomous Bilateral Teleoperation of Hexapod Robot Based on Haptic Force Feedback. J. Intell. Robot. Syst..

[5] Schmid K., Lutz P., Tomíc T., Mair E., Hirschmüller H. (2014). Autonomous vision-based microair vehicle for indoor and outdoor navigation. J. Field Robots.

[6] Gao Y., Liu S., Atia M.M., Noureldin A. (2015). INS/GPS/LiDAR Integrated Navigation System for Urban and Indoor Environments Using Hybrid Scan Matching Algorithm. Sensors.

[7] Shamsudin A.U., Ohno K., Hamada R., Kojima S., Westfechtel T., Suzuki T., Okada Y., Tadokoro S., Fujita J., Amano H. (2018). Consistent map building in petrochemical complexes for firefighter robots using SLAM based on GPS and LIDAR. Robomech. J..

[8] Abdelaziz N., El-Rabbany A. (2022). An Integrated INS/LiDAR SLAM Navigation System for GNSS-Challenging Environments. Sensors.

[9] Aboutaleb A., El-Wakeel A.S., Elghamrawy H., Noureldin A. (2020). LiDAR/RISS/GNSS dynamic integration for land vehicle robust positioning in challenging GNSS environments. Remote Sens..

[10] Zhao S., Fang Z., Li H., Scherer S. A Robust Laser-Inertial Odometry and Mapping Method for Large-Scale Highway Environments. Proceedings of the 2019 IEEE/RSJ International Conference on Intelligent Robots and Systems (IROS).

[11] Qin C., Ye H., Pranata C.E., Han J., Zhang S., Liu M. LINS: A Lidar-Inertial State Estimator for Robust and Efficient Navigation. Proceedings of the 2020 IEEE International Conference on Robotics and Automation (ICRA).

[12] Xu W., Zhang F. (2021). Fast-LIO: A fast, robust LiDAR-inertial odometry package by tightly-coupled iterated Kalman filter. IEEE Robots Autom. Lett..

[13] Xu W., Cai Y., He D., Lin J., Zhang F. (2022). Fast-lio2: Fast direct lidar-inertial odometry. IEEE Trans. Robots.

[14] Li J., You B., Ding L., Xu J., Li W., Chen H., Gao H. (2018). A Novel Bilateral Haptic Teleoperation Approach for Hexapod Robot Walking and Manipulating with Legs. Robot. Auton. Syst..

[15] Bai C., Xiao T., Chen Y., Wang H., Zhang F., Gao X. (2022). Faster-LIO: Lightweight Tightly Coupled Lidar-Inertial Odometry Using Parallel Sparse Incremental Voxels. IEEE Robots Autom. Lett..

[16] Chang L., Niu X., Liu T., Tang J., Qian C. (2019). GNSS/INS/LiDAR-SLAM Integrated Navigation System Based on Graph Optimization. Remote Sens..

[17] Kukko A., Kaijaluoto R., Kaartinen H., Lehtola V.V., Jaakkola A., Hyyppä J. (2017). Graph SLAM correction for single scanner MLS forest data under boreal forest canopy. Isprs J. Photogramm. Remote Sens..

[18] Hess W., Kohler D., Rapp H., Andor D. Real-time loop closure in 2D LIDAR SLAM. Proceedings of the IEEE International Conference Robotics and Automation.

[19] Chang L., Niu X., Liu T. (2020). GNSS/IMU/ODO/LiDAR-SLAM Integrated Navigation System Using IMU/ODO Pre-Integration. Sensors.

[20] Pierzchała M., Giguère P., Astrup R. (2018). Mapping forests using an unmanned ground vehicle with 3D LiDAR and graph-SLAM. Comput. Electron. Agric..

[21] Google Cartographer. https://google-cartographer.readthedocs.io/en/latest/.

[22] Zhang J., Singh S. (2017). Low-drift and Real-time Lidar Odometry and Mapping. Auton. Robot..

[23] Geiger A., Lenz P., Stiller C., Urtasun R. (2013). Vision meets robotics: The KITTI dataset. Int. J. Robots Res..

[24] Xue G., Wei J., Li R., Cheng J. (2022). LeGO-LOAM-SC: An Improved Simultaneous Localization and Mapping Method Fusing LeGO-LOAM and Scan Context for Underground Coalmine. Sensors.

[25] Oelsch M., Karimi M., Steinbach E. (2021). R-LOAM: Improving LiDAR odometry and mapping with point-to-mesh features of a known 3D Reference Object. IEEE Robots Autom. Lett..

[26] Wang H., Wang C., Chen C.L., Xie L. F-LOAM: Fast LiDAR odometry and mapping. Proceedings of the 2021 IEEE/RSJ International Conference Intelligent Robots and Systems (IROS) IEEE.

[27] Shan T., Englot B., Meyers D., Wang W., Ratti C., Rus D. LIO-SAM: Tightly-coupled LiDAR inertial odometry via smoothing and mapping. Proceedings of the 2020 IEEE/RSJ International Conference Intelligent Robots and Systems (IROS).

[28] Li T., Pei L., Xiang Y., Wu Q., Xia S., Tao L., Guan X., Yu W. (2021). P3-LOAM: PPP/LiDAR loosely coupled SLAM with accurate covariance estimation and robust RAIM in urban canyon environment. IEEE Sensors J..

[29] Kaess M., Johannsson H., Roberts R., Ila V., Leonard J., Dellaert F. iSAM2: Incremental smoothing and mapping with fluid relinearization and incremental variable reordering. Proceedings of the 2011 IEEE International Conference on Robotics and Automation.

[30] He J., Sun C., Zhang B., Wang P. (2021). Adaptive error-state Kalman filter for attitude determination on a moving platform. IEEE Trans. Instrum. Meas..

[31] Evo: Python Package for the Evaluation of Odometry and SLAM. https://github.com/MichaelGrupp/evo.

